# Cigarette Smoking as a Risk Factor for Tuberculosis in Adults: Epidemiology and Aspects of Disease Pathogenesis

**DOI:** 10.3390/pathogens13020151

**Published:** 2024-02-07

**Authors:** Charles Feldman, Annette J. Theron, Moloko C. Cholo, Ronald Anderson

**Affiliations:** 1 Department of Internal Medicine, Faculty of Health Sciences, University of the Witwatersrand, York Road, Parktown, Johannesburg 2193, South Africa; charles.feldman@wits.ac.za; 2 Department of Immunology, School of Medicine, Faculty of Health Sciences, University of Pretoria, Bophelo Road, Prinshof, Pretoria 0083, South Africa; atheron@up.ac.za (A.J.T.); moloko.cholo@up.ac.za (M.C.C.)

**Keywords:** alveolar macrophage, antibiotic resistance, biofilm, disease recurrence, disease severity, glycolytic reprogramming, *Mycobacterium tuberculosis*, nicotine, smoking

## Abstract

It has been noted by the World Health Organisation that cases of tuberculosis in 2022 globally numbered 10.6 million, resulting in 1.3 million deaths, such that TB is one of the infectious diseases causing the greatest morbidity and mortality worldwide. Since as early as 1918, there has been an ongoing debate as to the relationship between cigarette smoking and TB. However, numerous epidemiological studies, as well as meta-analyses, have indicated that both active and passive smoking are independent risk factors for TB infection, development of reactivation TB, progression of primary TB, increased severity of cavitary disease, and death from TB, among several other considerations. With this considerable body of evidence confirming the association between smoking and TB, it is not surprising that TB control programmes represent a key potential preventative intervention. In addition to coverage of the epidemiology of TB and its compelling causative link with smoking, the current review is also focused on evidence derived from clinical- and laboratory-based studies of disease pathogenesis, most prominently the protective anti-mycobacterial mechanisms of the alveolar macrophage, the primary intracellular refuge of *M. tuberculosis*. This section of the review is followed by an overview of the major strategies utilised by the pathogen to subvert these antimicrobial mechanisms in the airway, which are intensified by the suppressive effects of smoke inhalation on alveolar macrophage function. Finally, consideration is given to a somewhat under-explored, pro-infective activity of cigarette smoking, namely augmentation of antibiotic resistance due to direct effects of smoke per se on the pathogen. These include biofilm formation, induction of cellular efflux pumps, which eliminate both smoke-derived toxicants and antibiotics, as well as gene modifications that underpin antibiotic resistance.

## 1. Introduction

According to the WHO, in 2022, an estimated 10.6 million people fell ill with tuberculosis (TB) worldwide, with 1.3 million deaths [1]. Furthermore, a 2019 systematic review and meta-analysis indicated that the global prevalence of latent tuberculosis infection was 1 in 4 persons [2]. In several areas of the world, including South Africa, which has a particularly high incidence of TB, high prevalence rates of smoking and alcohol use have been reported among patients with TB [3]. Since as early as 1918 [4], there has been an ongoing debate as to the relationship between cigarette smoking and TB. An editorial in 2010 [5] suggested that numerous epidemiological studies as well as meta-analyses have indicated that both active and passive smoking are independent risk factors for TB infection (relative risk approximately 1.5–2), development of reactivation TB (relative risk approximately 2–3), progression of primary TB, increased severity of cavitary disease, and death from TB (relative risk approximately 1.5–3). While we acknowledge that there are negative studies, albeit very few, regarding smoking and at least some of the tuberculosis disease endpoints, these are mostly explained by various confounders [6,7]. With this considerable evidence of the association between smoking and TB, it has been recommended that TB control programmes must begin to address tobacco control as a potential preventative intervention [8].

This is a narrative review of publications in English accessed through PubMed and Google up until July 2023. The second section is focused on publications related to epidemiological aspects of smoking as a risk factor for development of TB. This is followed by an overview of the mechanisms utilised by alveolar macrophages to eradicate intracellular *Mycobacterium tuberculosis* organisms and the counter strategies used by the pathogen, as well as the harmful suppressive effects of smoking on macrophage-mediated anti-mycobacterial mechanisms. Finally, the direct effects of smoke on *M. tuberculosis* per se, which enable persistence of the pathogen in the airways, are given brief consideration.

## 2. Epidemiology of Smoking and Tuberculosis

Several older studies have confirmed, in various settings, that there is an association between smoking and TB [7,9,10,11,12,13,14,15,16]. In one of these studies, an increased TB risk was present among ever-smokers (odds ratio [OR] = 1.35; 95% CI 1.19 to1.53), current smokers (OR = 1.26; 95% CI 1.08 to 1.48), and past smokers (OR = 1.43; 95% CI 1.23 to 1.67) compared with never-smokers [16]. The risk for TB depends on the dose, intensity, duration, and type of tobacco smoked [7,16]. In several of the studies, a strong dose–response relationship (including quantity and duration) was noted between tobacco smoking and TB [6,8], although this was not shown in all studies [13]. In one study among the elderly in Hong Kong, there was a statistically significant dose–response relationship observed with respect to active TB and culture-confirmed TB [17]. Furthermore, an early qualitative systematic review and meta-analysis, despite limitations in the available data, showed a relationship between smoking and various clinical manifestations and treatment responses of TB disease, leading the authors to conclude that the association between smoking and TB disease appeared to be causal [12]. Another systematic review and meta-analysis indicated that there was evidence that smoking was a risk for TB infection as well as TB disease, but that it was not clear whether smoking caused additional mortality risk in persons who already have active TB [18]. Even in young people, cigarette smoking is a risk factor for pulmonary TB, with a dose–response relationship with the number of cigarettes consumed daily [19]. Thus, the bulk of the early literature indicated that the association between smoking and TB was substantial (without necessarily separating out the risk of transition from being exposed to being infected and that of being infected to development of TB disease) [20].

### 2.1. Passive Smoking

There is also significant evidence in the literature that passive smoking is a risk factor for TB [12,17,21,22]. For example, an early study indicated that passive exposure to tobacco smoke in children was associated with an increased risk of developing pulmonary TB immediately following infection [21]. Children aged 0–4 years and 5–9 years had a significantly higher risk than those 10 years of age and older. There was a dose–response risk of developing active TB immediately following infection according to the greater number of cigarettes smoked daily by the household adults (*p* < 0.001). Two systematic reviews and a meta-analysis also provided evidence of an association between passive smoking and TB disease [12,22]. In the one meta-analysis, the authors found that there was consistent evidence that cigarette smoking was positively associated with TB and, compared with those that did not smoke, smokers had an increased frequency of a positive tuberculin skin test, as well as having and dying from active TB, while passive smoking increased the risk for TB disease, although these associations were less strongly supported by the evidence [22]. Leung et al. indicated that passive smoking was associated with the development of both active TB (hazard ratio [HR] = 1.49; 95% CI 1.01 to 2.19) and culture-confirmed TB (HR = 1.70; 95% CI 1.04 to 2.80) on follow-up after potentially confounding background variables were controlled for [23].

### 2.2. Other Forms of Smoke Inhalation

It has also been noted that other forms of smoke inhalation, including beedi smoking [7,24], biomass fuel exposure [22], marijuana use [25], traffic-related air pollution, and possibly vaping [26], may be associated with risk of TB.

### 2.3. Clinical Aspects of Tuberculosis in Smokers

#### 2.3.1. Smoking Is Associated with a Delay in TB Diagnosis

There is considerable evidence that cigarette smoking is associated with a delay in the diagnosis of TB [27,28,29,30,31,32,33,34]. In a more recent study [34], smoking was significantly associated with patient-related diagnostic delay (adjusted odds ratio [AOR] = 81.8; 95% CI 3.67 to 19.56), although other studies, such as those mentioned above, have indicated that smoking is associated with both patient and health system delay. While all of the reasons for the delay in diagnosis are not fully known, symptoms such as cough due to TB not being recognised and being interpreted as being due to smoking may at least be partly responsible. The possible consequences of delayed diagnosis include more extensive disease, more complications, extended period of infectivity, higher mortality, increased rate of drug resistance in the community, increased need for referral for in-hospital care, and increased cost for both patients and the healthcare system [28].

#### 2.3.2. Smoking and Severity of TB Illness

There is also clear evidence that smoking increases the severity of TB [27,34,35,36,37,38,39], while the study by Adegbite mentioned above also documented a higher number of pulmonary TB signs and symptoms in smokers (AOR = 2.74; 95% CI 2.28 to 6.73) [34]. In one study, smokers developed more pulmonary disease (AOR = 1.5), more cavitary lung disease (AOR 1.9), and were more likely to need hospitalisation (AOR 1.8) that was more protracted [35]. Thus, in that study, smoking led to faster and more severe progression of TB. In addition, the cost of TB-related hospitalisation increases by approximately EUR 1,000,000 per year. Several other researchers have mentioned the impact of smoking on the severity of pulmonary lesions, including causation of more extensive lung lesions and lung cavitation [27,36,37,38].

#### 2.3.3. Influence of Smoking on Occurrence and Duration of Culture Positivity

Studies have shown that smoking is associated with more extensive positive smear and culture results at baseline [38]. One study of current smokers versus non-smokers indicated that former patients with TB had a 1.36-fold (95% CI 1.03–2.36) higher OR for culture-positive TB (*p* < 0.05) [37]. A very recent study reported a higher load of tubercle bacilli in sputum [39]. This was confirmed in an additional study in which the sputum mycobacterial load was significantly higher in smokers (AOR = 3.18; 95% CI 1.33 to 8.11) [34].

In addition to higher bacillary loads, several studies have indicated that cigarette smoking in patients with active TB is frequently associated with prolongation of smear positivity and/or culture positivity (culture non-conversion) at 60 days [36,37,38,40,41,42]. In one study, this delay in culture conversion was dose-dependent [42], with a significant trend of increasing AOR (*p* = 0.004) with greater smoking, rising to an AOR of 11.6 and 95% CI 1.8–73.4 in those smoking more than 20 cigarettes a day.

The concern with higher bacillary loads and prolongation of smear and/or culture positivity relates not only to the greater severity of illness but also to infectivity and duration of infectivity of the smoker with TB.

#### 2.3.4. Treatment Failure and Outcome of TB Treatment in Smokers

Smoking (and aging) has been shown to be associated with pulmonary TB treatment failure [43]. Even after adjustment for age, the risk of treatment failure was 2.1 times (95% CI 1.1 to 4.1) higher in patients who had a history of smoking. In another study, smoking was a major barrier to treatment success (OR = 0.76; 95% CI 0.69 to 0.84; *p* < 0.001) [44]. A more recent systematic review and meta-analysis confirmed a greater risk of poor treatment outcomes in smokers, indicating that smoking increased the likelihood of poor treatment outcomes by 51% (OR = 1.51; 95% CI 1.30 to 1.75) [45]. In one study, albeit in patients with multi-drug resistant TB, the group with a successful treatment outcome had a mean cigarette consumption of 14.7 ± 19.9 pack years, whereas in those with a poor outcome it was 40.5 ± 44.4 pack years (*p* = 0.0001) [46]. Smoking is the cause of half of the deaths of males with proven TB in India [47].

#### 2.3.5. Additional Outcomes Associated with TB Treatment in Smokers

Recurrence of disease after successful anti-TB therapy is significantly associated with smoking > 10 cigarettes a day [48]. Among patients with successful treatment completion, there was a clear gradient found (HR = 1.00, 1.33, 1.63) of relapse risk among never-smokers, ex-smokers, and current smokers, with a population attributable risk of 19.4% (12.2% in current smokers and 7.2% in ex-smokers) [36]. Chan and colleagues, as previously indicated, noted the numerous studies that indicated, among other risk factors regarding TB, that exposure to cigarette smoke was a risk for reactivation TB and progression of primary TB, suggesting, therefore, that cigarette smoke exposure may represent a criterion on which to base treatment of latent TB infection with an effective short course regimen, reinforced by programmes to reduce cigarette use [5].

Smoking is also clearly associated with treatment interruption, a negative effect on treatment completion, treatment default, and treatment loss to follow-up (OR = 1.35, 95% CI 1.21 to 1.50) [41], as well as treatment failure and death in smokers [38,41,44].

#### 2.3.6. Tobacco Control Programmes as an Essential Component of TB Control

Given the vast impact of cigarette smoking on TB disease, it is not surprising that for many years researchers have been stressing the essential need for smoking cessation strategies to control TB as part of TB control programmes [16,23,27,34,36,39,42,43,44,46,48]. Unfortunately, some studies have indicated that, at least in some areas, physicians’ knowledge, attitude, and practices of smoking cessation were limited [49]. Clearly in these areas, interventions are needed that target physician knowledge and skills to enable formalising tobacco dependence treatment within TB care. Healthcare workers also report structural characteristics that are primary barriers to offering behavioural support for smoking cessation, including high workloads and limited time spent with each patient, among other factors such as sociocultural barriers like gender relations, stigma, or social influences, which also need to be addressed [50]. Studies among healthcare workers have also led to the recognition that tobacco smoking cessation strategies should take place in communities as well as in healthcare facilities, supported by partnerships with media and families, while also addressing healthcare challenges to support effective implementation [51]. One follow-up study, undertaken 5 years after a smoking cessation intervention in Chinese TB patients, noted that non-smokers, ex-smokers, and current smokers who received the intervention maintained higher non-smoking rates than patients who did not [52]. The authors concluded that TB programme managers need to ensure integration and provision of smoking cessation advice and a smoke-free policy in routine TB services. A mathematical modelling study [53] noted that if smoking trends evident at the time of the study continued on the same trajectory, it was predicted that TB cases and related deaths would increase by 7% and 66%, respectively, while implementation of aggressive tobacco control measures could avert millions of deaths from TB.

While this section has concentrated primarily on cigarette smoking, it is important to remember that decreasing other forms of tobacco smoking, biomass fuel exposure, vaping, and exposure to traffic pollution would likely further reduce the impact on development, severity, and outcome of TB.

Key issues described in the preceding sections of this review are summarised in Table 1.

## 3. How Smoking Influences the Interaction of the Alveolar Macrophage with *M. tuberculosis*

As described in the preceding sections of this review, cigarette smoking represents a well-recognised and ominous risk factor for the development of TB, which is compounded by the antibiotic resilience of *M. tuberculosis* [54]. The mechanisms utilised by this pathogen to subvert the innate and adaptive protective immune mechanisms of the human and murine respiratory tracts, augmented by the immunosuppressive effects of inhalation of tobacco smoke, have been the subject of several recent comprehensive, informative reviews [55,56,57]. Taking this into account, the remaining sections of the current review are focused specifically on updating the mechanisms by which the pathogen partners with smoking to subvert the anti-mycobacterial activities of the alveolar macrophage, including the key involvement of nicotine in enabling intracellular bacterial survival, persistence, and proliferation, resulting in development of pulmonary TB. This approach necessitates an overview of the anti-mycobacterial mechanisms of alveolar macrophages.

### 3.1. Early Colonisation of the Respiratory Tract by M. tuberculosis

With respect to the earliest stages of disease pathogenesis in humans, inhalation of aerosolised *M. tuberculosis* organisms is normally countered by the expulsive actions of the respiratory mucociliary escalator. However, in smokers, the protective activities of this mechanism are compromised due to the ciliotoxic and cytotoxic effects induced by exposure of ciliated respiratory epithelium to the myriad of toxicants present in tobacco smoke, resulting in hypersecretion of dense, viscous airway mucus in the setting of decreased numbers of cilia, which exhibit slow and poorly coordinated ciliary beat frequency [58,59].

Having overcome entrapment and expulsion by the smoke-exposed, dysfunctional mucociliary escalator, the pathogen then progresses to the next and critical stage in disease pathogenesis, during which it takes refuge in its primary cellular host, the alveolar macrophage. Domiciled in this environment, *M. tuberculosis* per se possesses various mechanisms to promote intracellular survival and proliferation, which, as described in earlier studies, are augmented by exposure of airway macrophages to cigarette smoke [60,61]

### 3.2. Anti-Mycobacterial Mechanisms of the Alveolar Macrophage

*Mycobacterium tuberculosis* possesses a diverse array of pathogen-associated molecular patterns (PAMPs), many of which function as adhesins/invasins. These, in turn, promote recognition and internalisation of the pathogen enclosed in phagosomes via interaction with pattern recognition receptors (PRRs) expressed by alveolar macrophages [62,63]. These include: (i) Toll-like receptor 2 (TLR2) in its homodimeric and heterodimeric (TLR2/1, TLR2/6) forms as well as TLR4; (ii) the scavenger receptors CD36 (class B, member 3), class A macrophage scavenger receptors (SRA), macrophage receptor with a collagenous structure (MARCO), and C-type lectin receptors (DC-sign, Dectin-1, mannose receptor, and macrophage inducible C-type lectin known as mincle); and (iii) the lipopolysaccharide-interacting PRR, CD14. Other types of alveolar macrophage receptors include the complement receptors, CR1 and CR3, which bind the complement-derived opsonin, C3b, and CD43, which interacts with the chaperonin, Cpn 60.2, as well as receptors for pro-adhesive mycobacterial fimbriae [63,64].

In the absence of immunosuppression, the *M. tuberculosis*-containing phagosome undergoes a process of maturation associated with progressive acquisition of anti-mycobacterial activity. Phagosome maturation necessitates the recruitment of specific membrane-trafficking Rab GTPases and synthesis of phosphatidylinositol-3-phosphate (PI3P), which drive phagosome/lysosome fusion to form the phagolysosome, a membrane-bound organelle that is well equipped with an array of antimicrobial hydrolytic enzymes [65,66]. Completion of maturation results in intra-phagolysosomal acidification, decreasing to a pH of 4–5, which is mediated by recruitment of the vacuolar H^+^-ATPase. This, in turn, establishes a hostile antimicrobial environment endowed with bactericidal reactive oxygen species (ROS), reactive nitrogen species (RNS), and lysosomal proteolytic enzymes [65,66].

### 3.3. LC3-Associated Phagocytosis

Microtubule-associated protein 1A/1B light chain 3 (LC3)-associated phagocytosis, known as LC3-associated phagocytosis, represents an additional mechanism of macrophage-mediated intracellular elimination of *M. tuberculosis* [67,68,69]. This process, which involves elements of autophagy, recruits NADPH oxidase, NOX2, the activation of which enables delivery of LC3 to the outer membranes of single-membrane phagosomes to form laposomes. In turn, following enzymatic- and ROS-mediated lipidation of LC3, mature laposomes fuse with lysosomes to form phagolysosomes [68]. This event promotes bacterial killing by NOX2-derived ROS, both directly and indirectly, via intra-phagolysosome acidification as well as by structural stabilisation due to oxidation of membrane lipids.

### 3.4. Autophagy and Xenophagy

As described below, *M. tuberculosis* possesses an array of mechanisms to subvert phagosome and laposome maturation/acidification and lysosomal fusion of these pathogen-containing membrane-bound structures. As a strategy to bypass these pathogen-driven intracellular survival mechanisms, macrophages have developed an alternative bactericidal mechanism known as autophagy [70]. During this process, membrane permeabilisation of the dysfunctional phagosome occurs due to the action of the dedicated secretion system, ESAT-6 secretion system-1 (ESX-1), of *M. tuberculosis* and the membrane-disruptive effects of the major secretory protein, early secreted antigenic target-6 kDa (ESAT-6) [71]. This results in cytosolic translocation and exposure of bacterial DNA, recognised by the PRR known as STING (stimulator of interferon genes), which precedes ubiquitination of the pathogen. This event, in turn, marks *M. tuberculosis* for recognition by autophagy adaptors such as p62/sequestosome 1 (SQSTM1), which deliver the bacteria to autophagosomes that fuse with lysosomes to form autophagolysosomes [70,71,72]. Autophagosomes are derived from phagophores. These are double-membrane structures that undergo expansion and engulfment of cargo, followed by closure to form a sealed autophagosome.

Xenophagy is a process whereby autosomes capture ruptured immature phagosomes containing *M. tuberculosis* organisms. This is followed by the binding of cytoplasmic galectins 3, 8, and 9 to vacuolar glycans, promoting recruitment of the autophagy receptors, nuclear dot protein 52 (NDP52), calcium binding and coiled-coil domain 2 (CALCOCO2) and tripartite motif-containing (TRIM) proteins, which bind to LC3 and deliver contents to autophagosomes and also contribute to lysosome fusion [73].

Intracellular eradication of *M. tuberculosis* by the aforementioned mechanisms is preceded by reprogramming of macrophage energy metabolism, transitioning from predominantly oxidative phosphorylation to aerobic glycolysis. In this context, quiescent human alveolar macrophages, mainly of the M2 phenotype, isolated from bronchoalveolar lavage fluid have been found to undergo metabolic reprogramming to a pro-inflammatory, glycolytic phenotype. This process, which is driven by hypoxia-inducible factor (HIF-1α), results in the upregulation of genes encoding the glucose transporters, GLUT-1 and GLUT-6, and glycolytic enzymes including hexokinase-1, 6-phosphofructo-2-kinase/fructose-2,6-biphosphate, and lactate dehydrogenase [74,75,76,77]. This metabolic transition, which is triggered via interactions between mycobacterial PAMPs and alveolar macrophages [78], also results in glycolysis-related activation of the NLRP3 inflammasome-interleukin-1β-maturation and release pathway [79]. In addition, interactions of the glycolytic enzymes, aldolase and phosphofructokinase, are not only linked to provision of the ATP necessary to drive protons across the vacuolar membrane by the vacuolar H^+^-ATPase but also appear to stabilise this multi-component complex [80,81,82,83,84].

### 3.5. Mechanisms Used by M. tuberculosis to Subvert Macrophage Antimicrobial Activity

*Mycobacterium tuberculosis* utilises an array of proteins and lipids to subvert both macrophage phagocytosis and post-internalisation vacuolar maturation and fusion, which negatively affect all of the pathways mentioned above with respect to vacuolar acidification and eradication of the pathogen. Important examples of these *M. tuberculosis* survival mechanisms include the following:

- Impaired phagocytosis as a result of downregulation of the genes encoding the scavenger receptors, MARCO and collectin subfamily member-12 (COLEC12), albeit by mechanisms that remain to be established [85]. In addition, the mycobacterial lytR-CpsA-Psr (LCP) domain-containing cell wall protein, CpsA, has been reported to suppress LC3-associated phagocytosis and bacterial killing, the latter due to failure of recruitment of NOX2 to laposomes [86,87].
- Interference with vacuole biogenesis, phagosome maturation, and phagolysosome formation by various mechanisms that promote activation/antagonism of phosphatidylinositol-3-phosphate (PI3P), including PI3P secretory acid phosphatase (SapM), PI mannoside, and mannose-capped lipoarabinomannan, as well as inactivation of small Rab GTPases by mycobacterial nucleoside diphosphate kinase (Ndk) [55,70,88,89].
- Inhibition of the vacuolar H^+^-ATPase by secreted mycobacterial protein tyrosine phosphatase A (MptpA) [90].
- Induction of synthesis of microRNA-21 (miR-21) by isolated murine bone marrow-derived macrophages and human alveolar macrophages experimentally infected with *M. tuberculosis*, possibly due to interaction of the pathogen with TLR4, which, in turn, suppresses both glycolysis and expression of IL-1β [91]. The mechanism by which miR-21 suppresses glycolysis is linked to attenuation of expression/activity of the glycolytic enzyme, phosphofructokinase [91].
- With respect to inhibition of autophagy, mycobacterial sulphoglycolipids (SLS) and phthiocerol dimycocerosates (DIMs) have been reported to play a meaningful role [92]. In the case of the former, impairment of autophagy was associated with antagonism of TLR2-mediated activation of alveolar macrophages, while DIMs were found to attenuate the acidification of pathogen-containing laposomes [92]. In addition, the mycobacterium-derived terpenyl nucleoside, 1-tuberculosinyladnosine, has been reported to promote arrest of lysosomal maturation and autophagy blockage, resulting in transition of the macrophage from an M1 phenotype to an M2 foamy macrophage phenotype, which is “a cellular hallmark of tuberculosis disease” [93].

### 3.6. Impairment of Alveolar Macrophage Antimicrobial Activities by Inhalation of Cigarette Smoke

Smoking per se has been reported to interfere with the phagocytic activity of human alveolar macrophages as well as that of a human leukemia monocytic cell line (THP-1) following exposure to cigarette smoke extract in vitro, resulting from decreased expression of the PAMPs, TLR2 [94] and MARCO [95], respectively. An alternative mechanism of smoking-related impairment of macrophage phagocytosis has been described in patients with long-term cigarette smoking-related pulmonary TB [96]. In this context, isolated blood monocytes from long-term smoking TB patients were found to significantly increase levels of the microRNA, miR-196b-5p, relative to cells from non-smoking TB patients, while exposure of monocyte-derived macrophages to cigarette smoke extract was also associated with upregulation of expression of the miRNA [96]. Mechanistically, transfection of macrophages with miR-196b-5p resulted in “targeted inhibition” of suppressor of cytokine synthesis 3 (SOCS3) and activation of STAT3 (signal transducer and activator of transcription 3). These events were proposed to underpin the decreased uptake of the BCG vaccine strain of *M. bovis*, possibly as a consequence of attenuated synthesis of interleukin (IL)-6 and tumour necrosis factor alpha (TNFα) [96].

Notwithstanding the adverse effects on macrophage phagocytosis, smoking is also a prominent cause of defective post-phagocytic antimicrobial activity.

In this context, Monick et al. in an earlier study reported the existence of defective autophagy in alveolar macrophages isolated from the lungs of active smokers as well as in a murine macrophage cell line (Raw 246.7), following exposure to cigarette smoke extract in vitro [60]. Using a combination of transmission electron microscopy and sophisticated image analysis, the authors detected accumulation of autophagosomes and attenuation of autophagolysosome formation, consistent with cigarette smoke-mediated interference, with trafficking of the autophagosome to the lysosome [60]. Mechanistically, this appeared to result from failure of the recruitment of autophagy adaptors, which interact with ubiquitinated protein aggregates and bacterial targets to promote their association with the autophagosome membrane. This, in turn, resulted in failure of internalisation of the ubiquitinylated target, lysosomal fusion, and pathogen eradication [60].

More recently, smoking-mediated attenuation of post-phagocytic glycolytic reprogramming has emerged as a prominent mechanism of attenuation of macrophage antimicrobial activity against *M. tuberculosis* as well as other intracellular pathogens. In the case of *M. tuberculosis*, Gleeson et al. compared extracellular lactate concentrations, oxygen consumption and extracellular acidification rates, as well as expression of genes encoding glycolytic enzymes and the glucose transporter, GLUT-1, in human macrophages isolated from the lungs of smokers and non-smokers following induction of experimental infection with irradiated *M. tuberculosis* strain H37Rv [75]. At baseline, quiescent human alveolar macrophages exhibited higher rates of oxygen consumption and extracellular acidification than paired blood monocyte-derived macrophages. Alveolar macrophages investigated at baseline also demonstrated preferential metabolic utilisation of oxidative phosphorylation in the setting of a significant glycolytic reserve capacity. In contrast to cells from non-smokers, alveolar macrophages isolated from the lungs of smokers exhibited decreased oxygen consumption and extracellular acidification rates as well as a lesser glycolytic reserve at baseline [75]. Following induction of experimental infection with *M. tuberculosis*, smokers’ alveolar macrophages demonstrated lower acidification rates and antimicrobial lactate production [97], as well as attenuation of glycolytic reprogramming and expression of IL-1β and prostaglandin E2 [75]. Suppression of these activities in *M. tuberculosis*-infected smokers’ macrophages was also associated with decreased transcription of the *SCL2A1* gene, which encodes the glucose transporter, GLUT1, and genes encoding the glycolytic enzymes hexokinase 1,6-phophofructo-2-kinase/fructose-2,6-biphosphate and lactate dehydrogenase A, as well as the glycolysis-associated enzyme, pyruvate dehydrogenase kinase-3 [75]. The authors concluded that their research had identified “fundamental defects in the alveolar macrophage glycolytic response to infection after smoke exposure”, thereby compromising a critical innate host defence mechanism [75]. Although this interpretation of their data was justified, it is nevertheless somewhat surprising that the authors did not include an assay of macrophage anti-mycobacterial activity in their study.

### 3.7. Effects of Nicotine on the Anti-Mycobacterial Activity of Macrophages

Although the inhibitory effects of cigarette smoking on alveolar macrophage function are most likely attributable to the collective actions of the numerous toxicants present in smoke, Bai et al. identified nicotine as being a major contributor [98]. This was demonstrated using strategies that neutralised the cellular interactions of nicotine present in cigarette smoke extract: firstly, by treatment of isolated murine macrophages (alveolar or bone marrow-derived) or monocyte/macrophage cell lines with the nicotine acetylcholine receptor (nAChR) antagonist, mecamylamine; and secondly, by genetic disruption of various components of the nAChR [98]. As expected, exposure of *M. tuberculosis* (strain H37Rv)-infected murine macrophages to cigarette smoke extract significantly increased the number of intracellular bacilli, which was significantly attenuated by treatment of the cells with mecamylamine or by genetic disruption of nAChR, confirming the inhibitory effects of nicotine on macrophage anti-mycobacterial activity [98].

Mechanistically, treatment of a macrophage cell line (THP-1) or isolated murine alveolar macrophages with pure nicotine resulted in inhibition of autophagosome formation and increased numbers of intracellular bacilli, which were associated with inhibition of the transcription factor, NFκB, a positive regulator of autophagy [98]. In addition, nicotine induced production of the immunosuppressive cytokine, IL-10, and transforming growth factor-β by regulatory T cells (Tregs), which also contributed to the intracellular survival of *M. tuberculosis* [98]. Similar inhibitory effects of cigarette smoke extract-exposed Tregs on phagosome-lysosome and autophagosome formation by *M. tuberculosis*-infected human macrophages have been recently reported [99].

The aforementioned mechanisms by which cigarette smoking compromises alveolar macrophage anti-mycobacterial activity are summarised in Table 2.

## 4. Direct Pro-Infective Effects of Cigarette Smoke on *M. tuberculosis*

Evidence demonstrating the contribution of cigarette smoking to TB disease severity resulting from suppression of immune reactivity is widely available [100]. However, studies demonstrating the direct effects of smoking on the bacterium per se are very limited. We have previously shown that exposure of actively-replicating (AR) planktonic *M. tuberculosis* bacteria to cigarette smoke extract at concentrations lower than 3.12 mg/L had no effect on bacterial growth in vitro [101]. However, a study by Willemse et al. [102] described a non-lethal inhibitory effect of cigarette smoke extract on the growth of this bacterial population, achieving a higher minimum inhibitory concentration (MIC) of 125 mg/L. Similar inhibitory effects on mycobacterial growth were achieved in the presence of pure nicotine at the high concentration of 100 mg/L in vitro [98,103]. The findings on inhibition of bacterial growth by cigarette smoke extract were supported by gene expression data. These demonstrated that, at the sub-MIC level of 50 mg/L of smoke extract, the expression levels of the *Rv3727* gene, responsible for central metabolism [104], and the *Rv0341* gene, required for promotion of growth [105], were downregulated, indicating smoke-mediated inhibition of bacterial metabolism at the gene transcription level [102].

The requirement for relatively high concentrations of cigarette smoke extract to achieve inhibition of the growth of AR bacteria was attributed in part to high-level production of secreted anti-oxidative enzymes, including superoxide dismutase, catalase, and peroxidases by smoke-exposed organisms [101]. During short-term exposure of bacteria to smoke extract, 11 of the 59 upregulated genes constituted oxido-reductase enzymes, consistent with high-level oxidative stress [102]. The ferritin-encoding gene, *Rv3841 (brfB)*, required for bacterial growth [106], was also upregulated, possibly also related to oxidative stress due to the presence of iron in cigarette smoke [101].

Cigarette smoking has also been shown to increase the risk of antibiotic resistance in AR *M. tuberculosis* bacteria. In addition to a two-fold increase in the rate of development of resistance to rifampicin, several genes associated with resistance to various other antibiotics were differentially expressed [102]. These included the *Rv0678* gene, which encodes the MarR family of transcriptional regulators that control the transcriptional regulation of the MmpS5-MmpL5 efflux pump. Notably, several studies have shown that overexpression of MmpS5 and MmpL5 proteins reduced susceptibility to bedaquiline and clofazimine [107]. Another smoke-upregulated gene, *Rv3841* (*bfrB*), which, as mentioned above, encodes ferritin, also promoted resistance to the aminoglycosides, amikacin and kanamycin [106]. On the other hand, the *Rv3160c-Rv3161c* operon, encoding the TetR-like transcriptional repressor and a dioxygenase enzyme, respectively, which plays a role in lipid metabolism and cell wall biosynthesis, was downregulated following smoke exposure [102]. In this context, a previous study demonstrated that deletion of the *Rv3161c* gene led to increased resistance to isoniazid, implicating downregulation of this gene due to cigarette smoke exposure as a risk factor for antibiotic resistance [108]. Additionally, downregulation of the *Rv3727* gene, required for bacterial metabolism, and the *Rv0341* gene, required for promotion of growth in stressful conditions, supported the contention that alterations in bacterial metabolism, which promote development of dormancy, will also lead to drug tolerance in mycobacteria [102].

We have also observed that cigarette smoke exposure affects the development of biofilm by *M. tuberculosis*. However, unlike the situation with AR bacteria, the effects of cigarette smoke extract on biofilm were achieved at very low concentrations of the stressor (0.2–3.12 mg/L) [101]. During biofilm development, the environmental conditions of the pathogen change, becoming nutrient-limiting, hypoxic, and acidic, allowing the bacteria to transition from the AR phenotype to a non-replicating (NR) dormant, biofilm-forming phenotype [109,110]. Exposure of the biofilm-forming bacteria to smoke extract resulted in significantly increased biofilm biomass and attenuation of the decrease in the pH level, augmenting both bacterial persistence and antibiotic resistance [101]. In this context, acquisition of gene expression data will provide important mechanistic insights, which may impact drug design. These albeit somewhat limited in vitro studies provide evidence for how pathogen responses to cigarette smoke may result in changes in its behaviour that underpin dormancy, persistence, virulence, and antibiotic resistance, contributing to the clinical consequences described above. However, this contention needs to be confirmed in the clinical setting.

## 5. Conclusions

The susceptibility of smokers to development of severe, often prolonged, recurring and antibiotic-resistant TB is a longstanding, frustrating, and seemingly increasing global public health concern, with the major burden of disease having shifted to emergent nations. Often coupled with a lack of inclination to quit the smoking habit, this issue has largely been compounded by the ever-astute marketing strategies of the “big tobacco” companies via targeting young people in developing countries. Notwithstanding the abundance of compelling epidemiological evidence, clinical and laboratory studies have convincingly unravelled the mechanisms by which smoking contributes to the development, persistence, severity, and poor outcomes of TB; these are achieved not only by targeting the alveolar macrophage in particular but also the pathogen directly. In the case of the former, this is achieved via attenuation of various intracellular pathways involved in macrophage-mediated eradication of *M. tuberculosis*, and, in the latter, by induction of gene mutations that promote antibiotic resistance as well as by augmenting the formation of protective biofilm. Of additional concern, the seemingly prominent role of nicotine as a mediator of impaired macrophage antimicrobial activity casts doubt on the utility of nicotine replacement products as a meaningful strategy to counter the threat posed by *M. tuberculosis*.

## Figures and Tables

**Table 1:** The impact of cigarette smoking * on tuberculosis (TB) in smokers.

Condition
Increased risk of TB infection, active TB disease, and culture-confirmed TB.
Delay in TB diagnosis possibly leading to more extensive disease, more complications, extended period of infectivity, higher mortality, increased drug resistance in the community, increased need for referral for in-hospital management, and increased costs for the patient and the healthcare system.
Increased severity of TB with increase in pulmonary signs and symptoms, more extensive pulmonary disease, more lung cavitation, greater need for hospitalisation, and more prolonged hospitalisation.
Increased occurrence and duration of culture positivity, higher bacillary loads, and prolonged smear and culture positivity, possibly leading to greater severity of illness, increased infectivity, and duration of infectivity.
TB treatment failure, recurrence of disease after successful treatment with anti-TB drugs, increased progression of primary tuberculosis, reactivation of TB, and death.
Smoking is associated with treatment interruption, a negative effect on treatment completion, treatment default, treatment loss to follow-up, treatment failure, and death in smokers.

* There has also been some suggestion that passive smoking, as well as beedi smoking, marijuana use, vaping, indoor biomass fuel exposure, and traffic pollution exposure may also be associated with some of these negative effects in patients with TB who have these exposures. Data for this table were derived from cited articles [5,16,17,18,19,27,28,29,30,31,32,33,34,35,36,37,38,39,41,42,43,44,45,46,47,48].

**Table 2:** Mechanisms by which cigarette smoke and its nicotine constituent suppress alveolar macrophage anti-mycobacterial activity.

Impaired Alveolar Macrophage Function	Type of Study	Mechanism	References
Antimicrobial activity	Alveolar macrophages isolated from the lungs of smokers exposed to cigarette smoke extract in vitro	Attenuation of autophagolysosome formation due to failure of recruitment autophagy adaptors	[60]
Antimicrobial activity	Isolated lung macrophages from smokers exposed to *M. tuberculosis* in vitro	Failure of glycolytic reprogramming associated with decreased expression of genes encoding GLUT-1 as well as glycolysis-mediated activation of the NLRP3 inflammasome-IL-1β-maturation and release pathway	[75,79]
Phagocytosis	In vitro exposure of a macrophage cell line to cigarette smoke extract	Decreased expression of the PAMPs, TLR2 and MARCO	[94,95]
Phagocytosis	In vitro study using blood monocytes isolated from patients with long-term cigarette smoking-related active tuberculosis	Upregulated expression of the regulatory miRNA, mi-R-196b-5p, resulting in activation of suppressive STAT3	[96]
Antimicrobial activity	In vitro study involving smoke-exposed murine macrophages and macrophage cell lines depleted of nAChR or exposed to pure nicotine	Nicotine-mediated defective autophagosome formation due to inhibition of NFκB and activation of Tregs	[98,99]

Abbreviations: nAChR—nicotinic acetylcholine receptor; GLUT1—glucose transporter 1; MARCO—macrophage receptor with a collagenous structure; IL-1β—interleukin-1β; NFκB—nuclear factor kappa B; NLRP3 inflammasome—(nucleotide-binding domain, leucine-rich-containing family, pyrin domain-containing-3) inflammasome; miRNA—micro ribonucleic acid; PAMPs—pathogen-associated molecular patterns; STAT3—signal transducer and activation of transcription 3; TLR2—Toll-like receptor 2; Tregs—regulatory T cells.

## Data Availability

No applicable.

## References

[1] WHO (2023). Factsheet: Tuberculosis. https://www.who.int/news-room/fact-sheets/detail/tuberculosis#:~:text=Worldwide%2C%20TB%20is%20the%20second,all%20countries%20and%20age%20groups.

[2] Cohen A., Mathiasen V.D., Schön T., Wejse C. (2019). The global prevalence of latent tuberculosis: A systematic review and meta-analysis. Eur. Respir. J..

[3] Wessels J., Walsh C.M., Nel M. (2019). Smoking habits and alcohol use of patients with tuberculosis at Standerton Tuberculosis Specialised Hospital, Mpumalanga, South Africa. Health SA.

[4] Webb G.B. (1918). The effect of the inhalation of cigarette smoke on the lungs. A clinical study. Am. Rev. Tuberc..

[5] Chan E.D., Keane J., Iseman M.D. (2010). Should cigarette smoke exposure be a criterion to treat latent tuberculous infection?. Am. J. Respir. Crit. Care Med..

[6] Bay J.G., Patsche C.B., Svendsen N.M., Gomes V.F., Rudolf F., Wejse C. (2022). Tobacco smoking impact on tuberculosis treatment outcome: An observational study from West Africa. Int. J. Infect. Dis..

[7] Gambhir H.S., Kaushik R.M., Kaushik R., Sindhwani G. (2010). Tobacco smoking-associated risk for tuberculosis: A case-control study. Int. Health.

[8] Pai M., Mohan A., Dheda K., Leung C.C., Yew W.W., Christopher D.J., Sharma S.K. (2007). Lethal interaction: The colliding epidemics of tobacco and tuberculosis. Expert Rev. Anti Infect. Ther..

[9] Kolappan C., Gopi P.G. (2002). Tobacco smoking and pulmonary tuberculosis. Thorax.

[10] Den Boon S., van Lill S.W., Borgdorff M.W., Verver S., Bateman E.D., Lombard C.J., Enarson D.A., Beyers N. (2005). Association between smoking and tuberculosis infection: A population survey in a high tuberculosis incidence area. Thorax.

[11] Hassmiller K.M. (2006). The association between smoking and tuberculosis. Salud Publica Mex..

[12] Slama K., Chiang C.Y., Enarson D.A., Hassmiller K., Fanning A., Gupta P., Ray C. (2007). Tobacco and tuberculosis: A qualitative systematic review and meta-analysis. Int. J. Tuberc. Lung Dis..

[13] Alavi S.M., Ershadian S. (2009). Association between cigarette smoking and pulmonary tuberculosis. Pak. J. Med. Sci..

[14] Alavi-Naini R., Sharifi-Mood B., Metanat M. (2012). Association between tuberculosis and smoking. Int. J. High Risk Behav. Addict..

[15] Bishwakarma R., Kinney W.H., Honda J.R., Mya J., Strand M.J., Gangavelli A., Bai X., Ordway D.J., Iseman M.D., Chan E.D. (2015). Epidemiologic link between tuberculosis and cigarette/biomass smoke exposure: Limitations despite the vast literature. Respirology.

[16] Smith G.S., Van Den Eeden S.K., Baxter R., Shan J., Van Rie A., Herring A.H., Richardson D.B., Emch M., Gammon M.D. (2015). Cigarette smoking and pulmonary tuberculosis in northern California. J. Epidemiol. Community Health.

[17] Leung C.C., Li T., Lam T.H., Yew W.W., Law W.S., Tam C.M., Chan W.M., Chan C.K., Ho K.S., Chang K.C. (2004). Smoking and tuberculosis among the elderly in Hong Kong. Am. J. Respir. Crit Care Med..

[18] Bates M.N., Khalakdina A., Pai M., Chang L., Lessa F., Smith K.R. (2007). Risk of tuberculosis from exposure to tobacco smoke: A systematic review and meta-analysis. Arch. Intern. Med..

[19] Alcaide J., Altet M.N., Plans P., Parrón I., Folguera L., Saltó E., Domínguez A., Pardell H., Salleras L. (1996). Cigarette smoking as a risk factor for tuberculosis in young adults: A case-control study. Tuber. Lung Dis..

[20] Chiang C.Y., Slama K., Enarson D.A. (2007). Associations between tobacco and tuberculosis. Int. J. Tuberc. Lung Dis..

[21] Altet M.N., Alcaide J., Plans P., Taberner J.L., Saltó E., Folguera L.I., Salleras L. (1996). Passive smoking and risk of pulmonary tuberculosis in children immediately following infection. A case-control study. Tuber. Lung Dis..

[22] Lin H.H., Ezzati M., Murray M. (2007). Tobacco smoke, indoor air pollution and tuberculosis: A systematic review and meta-analysis. PLoS Med..

[23] Leung C.C., Lam T.H., Ho K.S., Yew W.W., Tam C.M., Chan W.M., Law W.S., Chan C.K., Chang K.C., Au K.F. (2010). Passive smoking and tuberculosis. Arch. Intern. Med..

[24] Tewatia P., Kaushik R.M., Kaushik R., Kumar S. (2020). Tobacco smoking as a risk factor for tuberculous pleural effusion: A case-control study. Glob. Health Epidemiol. Genom..

[25] Oeltmann J.E., Oren E., Haddad M.B., Lake L.k., Harrington T.A., Ijaz K., Narita M. (2006). Tuberculosis outbreak in marijuana users, Seattle, Washington, 2004. Emerg. Infect. Dis..

[26] Gómez A.C., Rodríguez-Fernández P., Villar-Hernández R., Gibert I., Muriel-Moreno B., Lacoma A., Prat-Aymerich C., Domínguez J. (2020). E-cigarettes: Effects in phagocytosis and cytokines response against *Mycobacterium tuberculosis*. PLoS ONE.

[27] Underner M., Perriot J. (2012). Tabac et tuberculose [Smoking and tuberculosis]. Presse Med..

[28] Gupta K.B., Bansal A. (2008). Delay in diagnosis and treatment of tuberculosis—A review. NTI Bull..

[29] Basnet R., Hinderaker S.G., Enarson D., Malla P., Mørkve O. (2009). Delay in the diagnosis of tuberculosis in Nepal. BMC Public Health.

[30] Shu W., Chen W., Zhu S., Hou Y., Mei J., Bai L., Xu W., Zhou L., Nie S., Cheng S. (2014). Factors causing delay of access to tuberculosis diagnosis among new, active tuberculosis patients: A prospective cohort study. Asia Pac. J. Public Health.

[31] Alavi S.M., Bakhtiyariniya P., Albagi A. (2015). Factors associated with delay in diagnosis and treatment of pulmonary tuberculosis. Jundishapur J. Microbiol..

[32] Das S., Basu M., Mandal A., Roy N., Chatterjee S., Dasgupta A. (2017). Prevalence and determinants of delay in diagnosis of pulmonary tuberculosis in Darjeeling district of West Bengal. J. Fam. Med. Prim. Care.

[33] Getnet F., Demissie M., Worku A., Gobena T., Tschopp R., Girmachew M., Assefa G., Seyoum B. (2019). Delay in diagnosis of pulmonary tuberculosis increases the risk of pulmonary cavitation in pastoralist setting of Ethiopia. BMC Pulm. Med..

[34] Adegbite B.R., Edoa J.R., Achimi Agbo P., Dejon-Agobé J.C., Essone P.N., Lotola-Mougeni F., Mbong Ngwese M., Mfoumbi A., Mevyann C., Epola M. (2020). Epidemiological, mycobacteriological, and clinical characteristics of smoking pulmonary tuberculosis patients, in Lambaréné, Gabon: A cross-sectional study. Am. J. Trop. Med. Hyg..

[35] Altet-Gômez M.N., Alcaide J., Godoy P., Romero M.A., Hernández del Rey I. (2005). Clinical and epidemiological aspects of smoking and tuberculosis: A study of 13,038 cases. Int. J. Tuberc. Lung Dis..

[36] Leung C.C., Yew W.W., Chan C.K., Chang K.C., Law W.S., Lee S.N., Tai L.B., Leung E.C., Au R.K., Huang S.S. (2015). Smoking adversely affects treatment response, outcome and relapse in tuberculosis. Eur. Respir. J..

[37] Chuang H.C., Su C.L., Liu H.C., Feng P.H., Lee K.Y., Chuang K.J., Lee C.N., Bien M.Y. (2015). Cigarette smoke is a risk factor for severity and treatment outcome in patients with culture-positive tuberculosis. Ther. Clin. Risk Manag..

[38] Mahishale V., Patil B., Lolly M., Eti A., Khan S. (2015). Prevalence of smoking and its impact on treatment outcomes in newly diagnosed pulmonary tuberculosis patients: A hospital-based prospective study. Chonnam Med. J..

[39] Feng Y., Xu Y., Yang Y., Yi G., Su H., Chen H., Guo R., Jia J., Wang P. (2023). Effects of smoking on the severity and transmission of pulmonary tuberculosis: A hospital-based case control study. Front. Public Health.

[40] Siddiqui U.A., O’Toole M., Kabir Z., Qureshi S., Gibbons N., Kane M., Keane J. (2010). Smoking prolongs the infectivity of patients with tuberculosis. Ir. Med. J..

[41] Wang E.Y., Arrazola R.A., Mathema B., Ahluwalia I.B., Mase S.R. (2020). The impact of smoking on tuberculosis treatment outcomes: A meta-analysis. Int. J. Tuberc. Lung Dis..

[42] Nijenbandring de Boer R., Oliveira e Souza Filho J.B., Cobelens F., Ramalho Dde P., Campino Miranda P.F., de Logo K., Oliveira H., Mesquita E., Oliveira M.M., Kritski A. (2014). Delayed culture conversion due to cigarette smoking in active pulmonary tuberculosis patients. Tuberculosis.

[43] Aguilar J.P., Arriaga M.B., Rodas M.N., Martins Netto E. (2019). Smoking and pulmonary tuberculosis treatment failure: A case-control study. J. Bras. Pneumol..

[44] Khan A.H., Sulaiman S.A.S., Hassali M.A., Khan K.U., Ming L.C., Mateen O., Ullah M.O. (2020). Effect of smoking on treatment outcome among tuberculosis patients in Malaysia; a multicenter study. BMC Public Health.

[45] Burusie A., Enquesilassie F., Addissie A., Dessalegn B., Lamaro T. (2020). Effect of smoking on tuberculosis treatment outcomes: A systematic review and meta-analysis. PLoS ONE.

[46] Pazarli P., Duman D.Y., Moçin Ö.Y., Karagöz T. (2013). The effect of smoking on treatment outcome of multidrug-resistant tuberculosis. Turk. Toraks. Derg. (Turk. Thorac. J.).

[47] Gajalakshmi V., Peto R., Kanaka T.S., Jha P. (2003). Smoking and mortality from tuberculosis and other diseases in India: Retrospective study of 43000 adult male deaths and 35000 controls. Lancet.

[48] Yen Y.F., Yen M.Y., Lin Y.S., Lin Y.P., Shih H.C., Li L.H., Chou P., Deng C.Y. (2014). Smoking increases risk of recurrence after successful anti-tuberculosis treatment: A population-based study. Int. J. Tuberc. Lung Dis..

[49] Harutyunyan A., Abrahamyan A., Grigoryan Z., Hayrumyan V., Truzyan N., Petrosyan V. (2020). Smoking cessation knowledge, attitude and practices among tuberculosis physicians: A qualitative study. Tob. Prev. Cessat..

[50] Boeckmann M., Warsi S., Noor M., Dogar O., Mustagfira E.H., Firoze F., Zahid R., Readshaw A., Siddiqi K., Kotz D. (2019). Health worker and patient views on implementation of smoking cessation in routine tuberculosis care. NPJ Prim. Care Respir. Med..

[51] Rutebemberwa E., Nyamurungi K., Joshi S., Olando Y., Mamudu H.M., Pack R.P. (2021). Health workers’ perceptions on where and how to integrate tobacco use cessation services into tuberculosis treatment; a qualitative exploratory study in Uganda. BMC Public Health.

[52] Lin Y., Dlodlo R.A., Shu Q., Lin H., Huang Q., Meng X., Zeng X., Chen Y., Xiao L. (2019). Outcomes of a smoking cessation intervention at follow-up after 5 years among tuberculosis patients in China. Tob. Induc. Dis..

[53] Basu S., Stuckler D., Bitton A., Glantz S.A. (2011). Projected effects of tobacco smoking on worldwide tuberculosis control: Mathematical modelling analysis. BMJ.

[54] Liu Q., Zhu J., Dulberger C.L., Stanley S., Wilson S., Chung E.S., Wang X., Culviner P., Liu Y.J., Hicks N.D. (2022). Tuberculosis treatment failure associated with evolution of antibiotic resilience. Science.

[55] Chandra P., Grigsby S.J., Philips J.A. (2022). Immune evasion and provocation by *Mycobacterium tuberculosis*. Nat. Rev. Microbiol..

[56] Lugg S.T., Scott A., Parekh D., Naidu B., Thickett D.R. (2022). Cigarette smoke exposure and alveolar macrophages: Mechanisms for lung disease. Thorax.

[57] Quan D.H., Kwong A.J., Hansbro P.M., Britton W.J. (2022). No smoke without fire: The impact of cigarette smoking on the immune control of tuberculosis. Eur. Respir. Rev..

[58] Heijink I.H., Brandenburg S.M., Postma D.S., van Oosterhout A.J. (2012). Cigarette smoke impairs airway epithelial barrier function and cell-cell contact recovery. Eur. Respir. J..

[59] Prasetyo A., Sadhana U., Budiman J. (2021). Nasal mucociliary clearance in smokers: A systematic review. Int. Arch. Otorhinolaryngol..

[60] Monick M.M., Powers L.S., Walters K., Lovan N., Zhang M., Gerke A., Hansdottir S., Hunninghake G.W. (2010). Identification of an autophagy defect in smokers’ alveolar macrophages. J. Immunol..

[61] Shang S., Ordway D., Henao-Tamayo M., Bai X., Oberley-Deegan R., Shanley C., Orme I.M., Case S., Minor M., Ackart D. (2011). Cigarette smoke increases susceptibility to tuberculosis–evidence from in vivo and in vitro models. J. Infect. Dis..

[62] Squeglia F., Ruggiero A., De Simone A., Berisio R. (2018). A structural overview of mycobacterial adhesins: Key biomarkers for diagnostics and therapeutics. Protein Sci..

[63] Vinod V., Vijayrajratnam S., Vasudevan A.K., Biswas R. (2020). The cell surface adhesins of *Mycobacterium tuberculosis*. Microbiol. Res..

[64] Stamm C.E., Collins A.C., Shiloh M.U. (2015). Sensing of *Mycobacterium tuberculosis* and consequences to both host and bacillus. Immunol. Rev..

[65] Schnettger L., Rodgers A., Repnik U., Lai R.P., Pei G., Verdoes M., Wilkinson R.J., Young D.B., Gutierrez M.G. (2017). A Rab20-dependent membrane trafficking pathway controls *M. tuberculosis* replication by regulating phagosome spaciousness and integrity. Cell Host Microbe.

[66] Maphasa R.E., Meyer M., Dube A. (2021). The macrophage response to *Mycobacterium tuberculosis* and opportunities for autophagy inducing nanomedicines for tuberculosis therapy. Front. Cell Infect. Microbiol..

[67] Upadhyay S., Philips J.A. (2019). LC3-associated phagocytosis: Host defense and microbial response. Curr. Opin. Immunol..

[68] Yuan J., Zhang Q., Chen S., Yan M., Yue L. (2022). LC3-Associated Phagocytosis in Bacterial Infection. Pathogens.

[69] Augenstreich J., Phan A.T., Allen C.N.S., Srinivasan L., Briken V. (2022). Spatio-temporal analysis of LC3 association to *Mycobacterium tuberculosis* phagosomes in human macrophages. bioRxiv.

[70] Deretic V. (2008). Autophagy, an immunologic magic bullet: *Mycobacterium tuberculosis* phagosome maturation block and how to bypass it. Future Microbiol..

[71] Bento C.F., Empadinhas N., Mendes V. (2015). Autophagy in the fight against tuberculosis. DNA Cell Biol..

[72] Fischer T.D., Wang C., Padman B.S., Lazarou M., Youle R.J. (2020). STING induces LC3B lipidation onto single-membrane vesicles via the V-ATPase and ATG16L1-WD40 domain. J. Cell Biol..

[73] Rubio-Tomás T., Sotiriou A., Tavernarakis N. (2023). The interplay between selective types of (macro)autophagy: Mitophagy and xenophagy. Int. Rev. Cell Mol. Biol..

[74] Shi L., Jiang Q., Bushkin Y., Subbian S., Tyagi S. (2019). Biphasic dynamics of macrophage immunometabolism during *Mycobacterium tuberculosis* infection. MBio.

[75] Gleeson L.E., O’Leary S.M., Ryan D., McLaughlin A.M., Sheedy F.J., Keane J. (2018). Cigarette smoking impairs the bioenergetic immune response to *Mycobacterium tuberculosis* infection. Am. J. Respir. Cell Mol. Biol..

[76] Wang T., Liu H., Lian G., Zhang S.Y., Wang X., Jiang C. (2017). HIF1α-induced glycolysis metabolism is essential to the activation of inflammatory macrophages. Mediat. Inflamm..

[77] Hackett E.E., Sheedy F.J. (2020). An army marches on its stomach: Metabolic intermediates as antimicrobial mediators in *Mycobacterium tuberculosis* infection. Front. Cell Infect Microbiol..

[78] Namgaladze D., Brüne B. (2023). Rapid glycolytic activation accompanying innate immune responses: Mechanisms and function. Front. Immunol..

[79] Yu Q., Guo M., Zeng W., Zeng M., Zhang X., Zhang Y., Zhang W., Jiang X., Yu B. (2022). Interactions between NLRP3 inflammasome and glycolysis in macrophages: New insights into chronic inflammation pathogenesis. Immun. Inflamm. Dis..

[80] Lu M., Sautin Y.Y., Holliday L.S., Gluck S.L. (2004). The glycolytic enzyme aldolase mediates assembly, expression, and activity of vacuolar H+-ATPase. J. Biol. Chem..

[81] Lu M., Ammar D., Ives H., Albrecht F., Gluck S.L. (2007). Physical interaction between aldolase and vacuolar H+-ATPase is essential for the assembly and activity of the proton pump. J. Biol. Chem..

[82] Zhao J., Beyrakhova K., Liu Y., Alvarez C.P., Bueler S.A., Xu L., Xu C., Boniecki M.T., Kanelis V., Luo Z.Q. (2017). Molecular basis for the binding and modulation of V-ATPase by a bacterial effector protein. PLoS Pathog..

[83] Gutiérrez S., Fischer J., Ganesan R., Hos N.J., Cildir G., Wolke M., Pessia A., Frommolt P., Desiderio V., Velagapudi V. (2021). *Salmonella typhimurium* impairs glycolysis-mediated acidification of phagosomes to evade macrophage defense. PLoS Pathog..

[84] Parra K.J., Chan C.Y., Chen J. (2014). *Saccharomyces cerevisiae* vacuolar H+-ATPase regulation by disassembly and reassembly: One structure and multiple signals. Eukaryot. Cell.

[85] Lavalett L., Rodriguez H., Ortega H., Sadee W., Schlesinger L.S., Barrera L.F. (2017). Alveolar macrophages from tuberculosis patients display an altered inflammatory gene expression profile. Tuberculosis.

[86] Wang Q., Zhu L., Jones V., Wang C., Hua Y., Shi X., Feng X., Jackson M., Niu C., Gao Q. (2015). CpsA, a LytR-CpsA-Psr family protein in *Mycobacterium marinum*, is required for cell wall integrity and virulence. Infect. Immun..

[87] Köster S., Upadhyay S., Chandra P., Papavinasasundaram K., Yang G., Hassan A., Grigsby S.J., Mittal E., Park H.S., Jones V. (2017). *Mycobacterium tuberculosis* is protected from NADPH oxidase and LC3-associated phagocytosis by the LCP protein CpsA. Proc. Natl. Acad. Sci. USA.

[88] Upadhyay S., Mittal E., Philips J.A. (2018). Tuberculosis and the art of macrophage manipulation. Pathog. Dis..

[89] Pal R., Bisht M.K., Mukhopadhyay S. (2022). Secretory proteins of *Mycobacterium tuberculosis* and their roles in modulation of host immune responses: Focus on therapeutic targets. FEBS J..

[90] Wong D., Bach H., Sun J., Hmama Z., Av-Gay Y. (2011). *Mycobacterium tuberculosis* protein tyrosine phosphatase (PtpA) excludes host vacuolar-H+-ATPase to inhibit phagosome acidification. Proc. Natl. Acad. Sci. USA.

[91] Hackett E.E., Charles-Messance H., O’Leary S.M., Gleeson L.E., Muñoz-Wolf N., Case S., Wedderburn A., Johnston D.G.W., Williams M.A., Smyth A. (2020). *Mycobacterium tuberculosis* limits host glycolysis and IL-1β by restriction of PFK-M via MicroRNA-21. Cell Rep..

[92] Bah A., Sanicas M., Nigou J., Guilhot C., Astarie-Dequeker C., Vergne I. (2020). The lipid virulence factors of *Mycobacterium tuberculosis* exert multilayered control over autophagy-related pathways in infected human macrophages. Cells.

[93] Bedard M., van der Niet S., Bernard E.M., Babunovic G., Cheng T.Y., Aylan B., Grootemaat A.E., Raman S., Botella L., Ishikawa E. (2023). A terpene nucleoside from *M. tuberculosis* induces lysosomal lipid storage in foamy macrophages. J. Clin. Investig..

[94] Droemann D., Goldmann T., Tiedje T., Zabel P., Dalhoff K., Schaaf B. (2005). Toll-like receptor 2 expression is decreased on alveolar macrophages in cigarette smokers and COPD patients. Respir. Res..

[95] Baqir M., Chen C.Z., Martin R.J., Thaikoottathil J., Case S.R., Minor M.N., Bowler R., Chu H.W. (2008). Cigarette smoke decreases MARCO expression in macrophages: Implication in *Mycoplasma pneumoniae* infection. Respir. Med..

[96] Yuan Y., Lin D., Feng L., Huang M., Yan H., Li Y., Chen Y., Lin B., Ma Y., Ye Z. (2018). Upregulation of miR-196b-5p attenuates BCG uptake via targeting SOCS3 and activating STAT3 in macrophages from patients with long-term cigarette smoking-related active pulmonary tuberculosis. J. Transl. Med..

[97] Ó Maoldomhnaigh C., Cox D.J., Phelan J.J., Mitermite M., Murphy D.M., Leisching G., Thong L., O’Leary S.M., Gogan K.M., McQuaid K. (2021). Lactate alters metabolism in human macrophages and improves their ability to kill *Mycobacterium tuberculosis*. Front. Immunol..

[98] Bai X., Stitzel J.A., Bai A., Zambrano C.A., Phillips M., Marrack P., Chan E.D. (2017). Nicotine impairs macrophage control of *Mycobacterium tuberculosis*. Am. J. Respir. Cell Mol. Biol..

[99] Bai X., Verma D., Garcia C., Musheyev A., Kim K., Fornis L., Griffith D.E., Li L., Whittel N., Gadwa J. (2023). Ex vivo and in vivo evidence that cigarette smoke-exposed T regulatory cells impair host immunity against *Mycobacterium tuberculosis*. Front. Cell. Infect. Microbiol..

[100] Miramontes C.V., Rodríguez-Carlos A., Marin-Luévano S.P., Trejo Martínez L.A., de Haro Acosta J., Enciso-Moreno J.A., Rivas-Santiago B. (2021). Nicotine promotes the intracellular growth of *Mycobacterium tuberculosis* in epithelial cells. Tuberculosis.

[101] Cholo M.C., Rasehlo S.S.M., Venter E., Venter C., Anderson R. (2020). Effects of cigarette smoke condensate on growth and biofilm formation by *Mycobacterium tuberculosis*. Biomed. Res. Int..

[102] Willemse D., Moodley C., Mehra S., Kaushal D. (2021). Transcriptional response of *Mycobacterium tuberculosis* to cigarette smoke condensate. Front. Microbiol..

[103] Greenstein R.J., Su L., Brown S.T. (2011). Growth of *M. avium* subspecies paratuberculosis in culture is enhanced by nicotinic acid, nicotinamide, and α and β nicotinamide adenine dinucleotide. Dig. Dis. Sci..

[104] Maxson M.E., Das L., Goldberg M.F., Porcelli S.A., Chan J., Jacobs W.R. (2023). *Mycobacterium tuberculosis* central metabolism is key regulator of macrophage pyroptosis and host immunity. Pathogens.

[105] Abdalla A.E., Yan S., Zeng J., Deng W., Xie L., Xie J. (2020). *Mycobacterium tuberculosis* Rv0341 promotes Mycobacterium survival in *in vitro* hostile environments and within macrophages and induces cytokines expression. Pathogens.

[106] Sharma D., Lata M., Faheem M., Khan A.U., Joshi B., Venkatesan K., Shukla S., Bisht D.M. (2016). *tuberculosis* ferritin (Rv3841): Potential involvement in Amikacin (AK) & Kanamycin (KM) resistance. Biochem. Biophys. Res. Commun..

[107] Radhakrishnan A., Kumar N., Wright C.C., Chou T.H., Tringides M.L., Bolla J.R., Lei H.T., Rajashankar K.R., Su C.C., Purdy G.E. (2014). Crystal structure of the transcriptional regulator Rv0678 of *Mycobacterium tuberculosis*. J. Biol. Chem..

[108] Tükenmez H., Sarkar S., Anoosheh S., Kruchanova A., Edström I., Harrison G.A., Stallings C.L., Almqvist F., Larsson C. (2021). *Mycobacterium tuberculosis* Rv3160c is a TetR-like transcriptional repressor that regulates expression of the putative oxygenase Rv3161c. Sci. Rep..

[109] Ojha A.K., Baughn A.D., Sambandan D., Hsu T., Trivelli X., Guerardel Y., Alahari A., Kremer L., Jacobs W.R., Hatfull G.F. (2008). Growth of *Mycobacterium tuberculosis* biofilms containing free mycolic acids and harbouring drug-tolerant bacteria. Mol. Microbiol..

[110] Mothiba M.T., Anderson R., Fourie B., Germishuizen W.A., Cholo M.C. (2015). Effects of clofazimine on planktonic and biofilm growth of *Mycobacterium tuberculosis* and *Mycobacterium smegmatis*. J. Glob. Antimicrob. Resist..

